# Supplementary material for: In vitro generation of functional murine heart organoids via FGF4 and extracellular matrix
Source: Nat Commun. 2020 Sep 3;11:4283. doi: 10.1038/s41467-020-18031-5 (PMC7471119; doi:10.1038/s41467-020-18031-5)
Supplement: Supplementary file 10 — Reporting Summary [file 41467_2020_18031_MOESM10_ESM.pdf]

nature research

Corresponding author(s): Jiyoung Lee, Fumitoshi Ishino

Last updated by author(s): Jul 13, 2020

Reporting Summary

Nature Research wishes to improve the reproducibility of the work that we publish. This form provides structure for consistency and transparency in reporting. For further information on Nature Research policies, see our Editorial Policies and the Editorial Policy Checklist.

Statistics

For all statistical analyses, confirm that the following items are present in the figure legend, table legend, main text, or Methods section.

n/a	Confirmed	
☐	☒	The exact sample size (n) for each experimental group/condition, given as a discrete number and unit of measurement
☐	☒	A statement on whether measurements were taken from distinct samples or whether the same sample was measured repeatedly
☐	☒	The statistical test(s) used AND whether they are one- or two-sided<br>*Only common tests should be described solely by name; describe more complex techniques in the Methods section.*
☒	☐	A description of all covariates tested
☐	☒	A description of any assumptions or corrections, such as tests of normality and adjustment for multiple comparisons
☐	☒	A full description of the statistical parameters including central tendency (e.g. means) or other basic estimates (e.g. regression coefficient) AND variation (e.g. standard deviation) or associated estimates of uncertainty (e.g. confidence intervals)
☐	☒	For null hypothesis testing, the test statistic (e.g. F, t, r) with confidence intervals, effect sizes, degrees of freedom and P value noted<br>*Give P values as exact values whenever suitable.*
☒	☐	For Bayesian analysis, information on the choice of priors and Markov chain Monte Carlo settings
☒	☐	For hierarchical and complex designs, identification of the appropriate level for tests and full reporting of outcomes
☒	☐	Estimates of effect sizes (e.g. Cohen's d, Pearson's r), indicating how they were calculated

Our web collection on statistics for biologists contains articles on many of the points above.

Software and code

Policy information about availability of computer code

Data collection	No customized software was used.
Data analysis	Optical mapping: BV analysis software (Brainvision)<br>Ca2+ imaging: ImageJ software, Origin software (OriginLab)<br>Graphs in Fig. 10b, c and Supplementary Figure 2a: GraphPad Prism version 8<br>Immunofluorescence: ZEN 2012 SP1 (black edition) version 8.1

For manuscripts utilizing custom algorithms or software that are central to the research but not yet described in published literature, software must be made available to editors and reviewers. We strongly encourage code deposition in a community repository (e.g. GitHub). See the Nature Research guidelines for submitting code & software for further information.

Data

Policy information about availability of data

All manuscripts must include a data availability statement. This statement should provide the following information, where applicable:

- Accession codes, unique identifiers, or web links for publicly available datasets
- A list of figures that have associated raw data
- A description of any restrictions on data availability

The accession codes of the raw sequencing data from the RNA-seq analyses are GSE143932 (GEO) [https://www.ncbi.nlm.nih.gov/geo/query/acc.cgi?acc=GSE143932] and SAMD00202559-SAMD00202583 (DDBJ BioSample) [http://trace.ddbj.nig.ac.jp/BSSearch/biosample?acc=SAMD00202559]-[http://trace.ddbj.nig.ac.jp/BSSearch/biosample?acc=SAMD00202583].
All relevant data are available from the authors upon reasonable request. Source data (Fig. 7a-e, 8d, Supplementary Figure 5c, 6b) are provided as a Source Data file.

Field-specific reporting

Please select the one below that is the best fit for your research. If you are not sure, read the appropriate sections before making your selection.

☒ Life sciences ☐ Behavioural & social sciences ☐ Ecological, evolutionary & environmental sciences

For a reference copy of the document with all sections, see nature.com/documents/nr-reporting-summary-flat.pdf

Life sciences study design

All studies must disclose on these points even when the disclosure is negative.

Sample size	On the generation of heart organoids, the sample size was determined based on the data collection of six independent experiments. The data were collected on the entire population of those experiments. Given that heart organoids were consistently generated in those experiments, the sample size of our study is sufficient.
Data exclusions	No data were excluded from the analyses.
Replication	The generation of heart organoids were performed over 10 experiments and successfully replicated . From different cell lines, the generation of heart organoids were reliably reproduced. All other results reported were successfully replicated. We have performed at least two independent or biologically independent experiments. We described the number of experiments in Figure legends.
Randomization	For the generation of heart organoids, EBs were randomly divided into control (+FGF4 in the standard condition) and several treatment samples (+FGF10, +FGF2, or Matrigel culture conditions).
Blinding	For generation of heart organoids, culturing EBs derived from murine ES cells with different conditions was performed in a non-blinded manner.

Reporting for specific materials, systems and methods

We require information from authors about some types of materials, experimental systems and methods used in many studies. Here, indicate whether each material, system or method listed is relevant to your study. If you are not sure if a list item applies to your research, read the appropriate section before selecting a response.

Materials & experimental systems

n/a	Involved in the study	
☐	☒	Antibodies
☐	☒	Eukaryotic cell lines
☒	☐	Palaeontology and archaeology
☐	☒	Animals and other organisms
☒	☐	Human research participants
☒	☐	Clinical data
☒	☐	Dual use research of concern

Methods

n/a	Involved in the study	
☒	☐	ChIP-seq
☒	☐	Flow cytometry
☒	☐	MRI-based neuroimaging

Antibodies

Antibodies used	anti-Tbx5 (Abcam, ab137833), anti-cTnI (Abcam, ab47003), anti-cTnT (Abcam, ab8295), anti-Nkx2-5 (Abcam, ab91196), anti-Nestin (Abcam, ab105389), anti-Oct3/4 (Santa Cruz Biotech, sc-8629), anti-PECAM (BD, #550274), anti-Mlc2a (Synaptic System #311 011), anti-Mlc2v (Synaptic System #310 003), anti-SM-MHC (R&D Systems, MAB4470), anti-αSMA (Abcam, ab5694), anti-TRPM4 (ABN418), anti-KCNN4 (IK1, GTX54786), anti-GATA4 (Abcam, ab134057), anti-AFP (Abcam, ab213328), anti-Cx43 (Sigma, C6219), anti-Cx40

(Invitrogen, #37-8900), anti-Cx45 (Abcam, ab78408) and anti-RYR (Abcam, ab2868).
Alexa 488 goat anti mouse IgG1 (Invitrogen, A21121), Alexa 568 goat anti rabbit IgG(H+L) (Invitrogen, A11036), Alexa 568 goat anti rat IgG(H+L) (Invitrogen, A11077), Alexa 488 goat anti rabbit IgG (Invitrogen, A11034), Goat anti mouse IgG2b Secondary Ab Alexa Fluor 647 (Invitrogen, A21242), Alexa Fluor 488 goat anti mouse IgG Ab (Invitrogen, A11001), Alexa 594 donkey anti goat IgG(H+L) (Invitrogen, A11058), Alexa 488 donkey anti rabbit IgG(H+L) (Invitrogen, A32790).

Validation

anti-Tbx5 (Abcam, ab137833): Suitable for WB, ICC/IF of human. Validated for detecting Tbx5 in mouse hearts.
anti-cTnI (Abcam, ab47003): Recommended for ICC/IF of mouse, rat and human. Validated for detecting cardiomyocytes in mouse hearts.
anti-cTnT (Abcam, ab8295): Recommended for Flow Cyt, IHC-Fr of mouse, rat, dog, and human. Validated for detecting cardiomyocytes in mouse hearts.
anti-Nkx2-5 (Abcam, ab91196): Recommended for ICC/IF of mouse, rat and human. Validated for detecting Nkx2.5 in mouse hearts.
anti-Nestin (Abcam, ab105389): Recommended for ICC/IF of human. Validated for detecting Nestin in mouse embryonic hearts.
anti-Oct3/4 (Santa Cruz Biotech, sc-8629): Recommended for WB, ChiP of mouse, rat and human. Validated for detecting Oct3/4 in mouse embryonic stem cells and EBs by ICC/IF.
anti-PECAM (BD, #550274): Recommended for Flow Cyt, IHC-Fr of mouse (QC Testing). Validated for detecting endothelial cells in mouse hearts.
anti-Mlc2a (Synaptic System #311 011): Recommended for IHC of human, mouse and rat. Validated for detecting atrial cells in mouse hearts.
anti-Mlc2v (Synaptic System #310 003): Recommended for IHC of human, mouse and rat. Validated for detecting ventricular cells in mouse hearts.
anti-SM-MHC (R&D Systems, MAB4470): Recommended for ICC, IHC of human and mouse. Validated for detecting smooth muscle cells in mouse hearts.
anti-αSMA (Abcam, ab5694): Recommended for IHC-FoFr, ICC/IF of mouse, rat and chicken. Validated for detecting smooth muscle cells in mouse hearts.
anti-TRPM4 (ABN418): Recommended for WB, IHC of human, mouse and rat. Validated for detecting Purkinje cells in mouse hearts.
anti-KCNN4 (IK1, GTX54786): Recommended for WB of human. Validated for detecting IK1 in mouse hearts by ICC/IF.
anti-GATA4 (Abcam, ab134057): Recommended for WB, Flow Cyt, ICC/IF of mouse, rat and human. Validated for detecting GATA4 in mouse hearts.
anti-AFP (Abcam, ab213328): Recommended for ICC/IF of mouse liver and placenta.
anti-Cx43 (Sigma, C6219): Recommended for IHC/Fr of human and mammals. Validated for detecting Cx43 (gap junction protein) in mouse hearts.
anti-Cx40 (Invitrogen, #37-8900): Recommended for IHC/Fr of human and mouse. Validated for detecting Cx40 (gap junction protein) in mouse hearts.
anti-Cx45 (Abcam, ab78408): Recommended for ICC of rat and predicted reactivity with mouse. Validated for detecting Cx45 (gap junction protein) in mouse hearts.
anti-RYR (Abcam, ab2868): Recommended for ICC/IF of mouse and rat. Validated for detecting Ryanodine receptor in mouse hearts.

Eukaryotic cell lines

Policy information about cell lines

Cell line source(s)	Mouse embryonic stem cell lines
Authentication	Given that all cell lines were originally established, none of cell lines have authenticated. If necessary, we will perform STR profiling for the cell line authentication.
Mycoplasma contamination	The cell lines were not tested for mycoplasma contamination. If necessary, we will test.
Commonly misidentified lines (See ICLAC register)	No commonly misidentified cell lines were used.

Animals and other organisms

Policy information about studies involving animals; ARRIVE guidelines recommended for reporting animal research

Laboratory animals	Pregnant female mice C57BL/6 (over 9 Weeks) from 9.5 to 13.5 days post coitum, postnatal day1 mice C57BL/6, and ES cell lines derived from mice C57BL/6 (over 9 Weeks) and F1 hybrid of C57BL/6 and JF1(over 9 Weeks) were used in this study.<br>Mice were allowed access to a standard chow diet and water ad libitum and were housed in a pathogen-free barrier facility with a 12L:12D cycle, at temperature and humidity ranges of 22 to 24°C and 40 to 60%, respectively.
Wild animals	The study did not involve wild animals.
Field-collected samples	The study did not involve field-collected samples.
Ethics oversight	All animal experiments were approved by the Institutional Animal Care and Use Committee of Tokyo Medical and Dental University (TMDU).

Note that full information on the approval of the study protocol must also be provided in the manuscript.
