# Supplementary material for: In vitro generation of functional murine heart organoids via FGF4 and extracellular matrix
Source: Nat Commun. 2020 Sep 3;11:4283. doi: 10.1038/s41467-020-18031-5 (PMC7471119; doi:10.1038/s41467-020-18031-5)
Supplement: Supplementary file 12 — Descriptions of Additional Supplementary Files [file 41467_2020_18031_MOESM12_ESM.pdf]

Title: Supplementary Movie 1.
Description: Spontaneous beating of *in vitro* cultured, multichambered heart organoid at day 11. QuickTime movie.

Title: Supplementary Movie 2.
Description: Spontaneous beating of *in vitro* cultured cardiac crescent-like structure at day 3. QuickTime movie.

Title: Supplementary Movie 3
Description: Time lapse from day 2 to day 8 of heart organoid culture showed that *in vitro* cultured EB underwent morphological changes to form a cardiac crescent-like structure, a heart tube and a looping heart tube. Scale bar, 100μm. MP4 file.

Title: Supplementary Movie 4.
Description: 3D imaging of Mlc2a (green) and Mlc2v (red) immunostained heart organoid cultured for 13 days. See also Figure 8b. MP4 file.

Title: Supplementary Movie 5.
Description: Calcium transients in cultured heart organoid at day 10.
For the analysis of calcium oscillation in heart organoid, the heart organoid was stained with the calcium-binding fluorescent dyes Fluo8 AM or Fluo8 AM/F127. The fluorescent calcium signal was recorded at 1/100 seconds of exposure time under fluorescence microscopy. QuickTime movie.

Title: Supplementary Movie 6.
Description: Propagation map of a cultured heart organoid at day 11. The atrium-like propagation before ventricle activation was detected by optical mapping. MP4 file.

Title: Supplementary Movie 7.
Description: Optical mapping of the *in vitro* cultured heart organoid at day 17, showing simultaneous excitement before administration of E4031 (1–10 seconds) and spontaneous induction of tachyarrhythmia after administration of E4031 (11–20 seconds). QuickTime movie. Two Movie files (10 seconds each) were combined into one MP4 file.

Title: Supplementary Data 1.
Description: List of genes differentially expressed genes between in vivo embryonic hearts (E9.5 and E11.5) and EBs.
