# Supplementary material for: In vitro generation of functional murine heart organoids via FGF4 and extracellular matrix
Source: Nat Commun. 2020 Sep 3;11:4283. doi: 10.1038/s41467-020-18031-5 (PMC7471119; doi:10.1038/s41467-020-18031-5)
Supplement: Supplementary file 13 — Source Data [file 41467_2020_18031_MOESM13_ESM.zip › Source Data/figure_console_text.rtf]

Figure 7A#PcaHO_RPKM <- HOrnaseq[,c(1,3,8,13,23,28,33,83,88,93,98,103,108,113,118)]   #EB, E9.5, E11.5 day9, day13 hbgenes <- c("Hbb-bh1","Hba-a2","Hbb-b1","Hbb-y","Hba-a1","Hbb-bt","Hbb-bs","Hbb-b2","Hba-x","Hp") �”hb complexŠÖ˜Aˆâ“`Žqpca_wohb <- prcomp(HO_RPKM[!HO_RPKM$geneName %in% hbgenes,2:15], scale=TRUE)library("rgl")plot3d(pca_wohb$rotation[,1:3], col=c("red","red","red","blue","blue","blue","green","green","green","darkgreen","darkgreen","darkgreen","orange","orange"), size=5)B#heatmapplot(regHeatmap(mat_logRPKM[,c(4,5,6,9,7,8,10,11,12,13,14,1,2,3)], dendrogram=list(Col=list(status="no")), labels=list(Row=list(cex=0.6))))C #dot plotgroup1 <- c("Hcn4","Hey2","Irx4","Myl2","Myl7","Tbx5")group2 <- c("Gata4","Actc1","Myh7","Bmp10","Pitx2","Nkx2-5","Tnnt2","Nppa")group3 <- c("Mesp1","Nanog","Nodal","Pou5f1","Wnt3")group4 <- c("Myh6","Tnnt3")group5 <- c("Foxa2","Myod1","Otx2","Pdx1","Sox17","Tbr1")iggplot(subset(HO_RPKM_L, geneName %in% group1), aes(x=factor, y=log2RPKM, colour=factor))+geom_jitter(width=0.2)+facet_wrap(~geneName, scale="free_y", nrow=1)+scale_colour_manual(values=c("orange","red","blue","green","darkgreen"))+theme_classic()+theme(axis.line=element_line(lineend="square",size=0.8), axis.text.x=element_text(angle=90,hjust=0, vjust=0.5, colour="black"), legend.position="none", axis.text.y=element_text(colour="black"), strip.text=element_text(face="italic"))+labs(x="",y="log2(RPKM+1)")iiggplot(subset(HO_RPKM_L, geneName %in% group2), aes(x=factor, y=log2RPKM, colour=factor))+geom_jitter(width=0.2)+facet_wrap(~geneName, scale="free_y", nrow=2)+scale_colour_manual(values=c("orange","red","blue","green","darkgreen"))+theme_classic()+theme(axis.line=element_line(lineend="square",size=0.8), axis.text.x=element_text(angle=90,hjust=0, vjust=0.5, colour="black"), legend.position="none", axis.text.y=element_text(colour="black"), strip.text=element_text(face="italic"))+labs(x="",y="log2(RPKM+1)")iiiggplot(subset(HO_RPKM_L, geneName %in% group3), aes(x=factor, y=log2RPKM, colour=factor))+geom_jitter(width=0.2)+facet_wrap(~geneName, scale="free_y", nrow=1)+scale_colour_manual(values=c("orange","red","blue","green","darkgreen"))+theme_classic()+theme(axis.line=element_line(lineend="square",size=0.8), axis.text.x=element_text(angle=90,hjust=0, vjust=0.5, colour="black"), legend.position="none", axis.text.y=element_text(colour="black"), strip.text=element_text(face="italic"))+labs(x="",y="log2(RPKM+1)")ivggplot(subset(HO_RPKM_L, geneName %in% group4), aes(x=factor, y=log2RPKM, colour=factor))+geom_jitter(width=0.2)+facet_wrap(~geneName, scale="free_y", nrow=2)+scale_colour_manual(values=c("orange","red","blue","green","darkgreen"))+theme_classic()+theme(axis.line=element_line(lineend="square",size=0.8), axis.text.x=element_text(angle=90,hjust=0, vjust=0.5, colour="black"), legend.position="none", axis.text.y=element_text(colour="black"), strip.text=element_text(face="italic"))+labs(x="",y="log2(RPKM+1)")vggplot(subset(HO_RPKM_L, geneName %in% group5), aes(x=factor, y=log2RPKM, colour=factor))+geom_jitter(width=0.2)+facet_wrap(~geneName, scale="free_y", nrow=2)+scale_colour_manual(values=c("orange","red","blue","green","darkgreen"))+theme_classic()+theme(axis.line=element_line(lineend="square",size=0.8), axis.text.x=element_text(angle=90,hjust=0, vjust=0.5, colour="black"), legend.position="none", axis.text.y=element_text(colour="black"), strip.text=element_text(face="italic"))+labs(x="",y="log2(RPKM+1)")Dupdowngenes <- c("Tnni3","Myl2","Myh6","Tnnt2","Myh7","Actc1","Nanog","Fgf8","Pou5f1","Mesp1")ggplot(resdf, aes(x=log2(baseMean), y=log2FoldChange, colour=col))+geom_point(size=0.3, aes(colour=col))+scale_colour_manual(values=c("black","blue","lightpink","red","skyblue"))+labs(x="A",y="M",title="Heart Organoid vs EB")+theme_classic()+theme(axis.line=element_line(size=1, lineend="square"), axis.text=element_text(size=10, colour="black"), legend.position="none")+geom_text_repel(data=resdf[resdf$geneName %in% updowngenes,], aes(x=log2(baseMean), y=log2FoldChange,label=geneName), colour="black", force=10, min.segment.length = 0.001, fontface="italic")Eggplot(GO_top30, aes(x=reorder(GO.biological.process.complete, FDR), y=FDR))+geom_bar(fill="red",stat="identity")+coord_flip()+theme_classic()+theme(panel.background=element_rect(colour="black",size=1), axis.line=element_blank(), axis.text=element_text(colour="black"))+labs(x="",y="-log10(FDR)")ggplot(GO_low30, aes(x=reorder(GO.biological.process.complete, FDR), y=FDR))+geom_bar(fill="blue",stat="identity")+coord_flip()+theme_classic()+theme(panel.background=element_rect(colour="black",size=1), axis.line=element_blank(), axis.text=element_text(colour="black"))+labs(x="",y="-log10(FDR)")Supplementary Figure 5CVenous markersggplot(subset(artery_venous_RPKM,geneName %in% c("Nr2f2","Aplnr","Dab2","Tek")), aes(x=group, y=logrpkm, colour=group))+geom_jitter(width=0.2)+facet_wrap(~geneName, scales="free_y", nrow=1)+labs(x="",y="log2(RPKM + 1)", title="Venous markers", colour="")+theme(axis.text.x=element_text(angle=60, hjust=1))Arterial markersggplot(subset(artery_venous_RPKM,geneName %in% c("Cxcr4","Jag2","Msx1","Notch4","Dll4","Gja4")), aes(x=group, y=logrpkm, colour=group))+geom_jitter(width=0.2)+facet_wrap(~geneName, scales="free_y", nrow=2)+labs(x="",y="log2(RPKM + 1)", title="Arterial markers", colour="")+theme(axis.text.x=element_text(angle=60, hjust=1))Figure 8D#dot plot ggplot(subset(df_l, Name %in% c("Cend1","Col6a2","Cspg4","Fam189a2","Gstm6","Lrig1","Mb","Sema3c","Slc7a7","Snai3","Trim47","Gja1")), aes(x=factor, y=log2(FPKM + 1), fill=factor)) + geom_jitter(width=0.2, pch=21, colour="black")+ facet_wrap(~Name)+labs(fill="",x="")+theme(axis.text.x=element_text(angle=30, hjust=1))+stat_summary(data.fun="mean_se", geom="point", pch="-", size=7, show.legend=FALSE)Supplementary Figure 6B# mean log2 FPKM barplot top100 genes ggplot(l_df, aes(x=Name, y=meanFPKM, fill=aorv))+geom_bar(stat="identity", position="dodge")+facet_wrap(~type, nrow=2, labeller=as_labeller(c("E11.5"="E11.5_H","HO"="HO")))+theme(axis.text.x=element_text(angle=60,hjust=1), axis.text=element_text(colour="black"))+scale_fill_manual(values=c("#00BFC4","#F8766D"),name="")+labs(x="",y="mean log2(FPKM)")
