# Supplementary material for: In vitro generation of functional murine heart organoids via FGF4 and extracellular matrix
Source: Nat Commun. 2020 Sep 3;11:4283. doi: 10.1038/s41467-020-18031-5 (PMC7471119; doi:10.1038/s41467-020-18031-5)
Supplement: Supplementary file 8 — Supplementary Information [file 41467_2020_18031_MOESM8_ESM.pdf]

# *In vitro* generation of functional murine heart organoids via FGF4 and extracellular matrix

Lee et al.

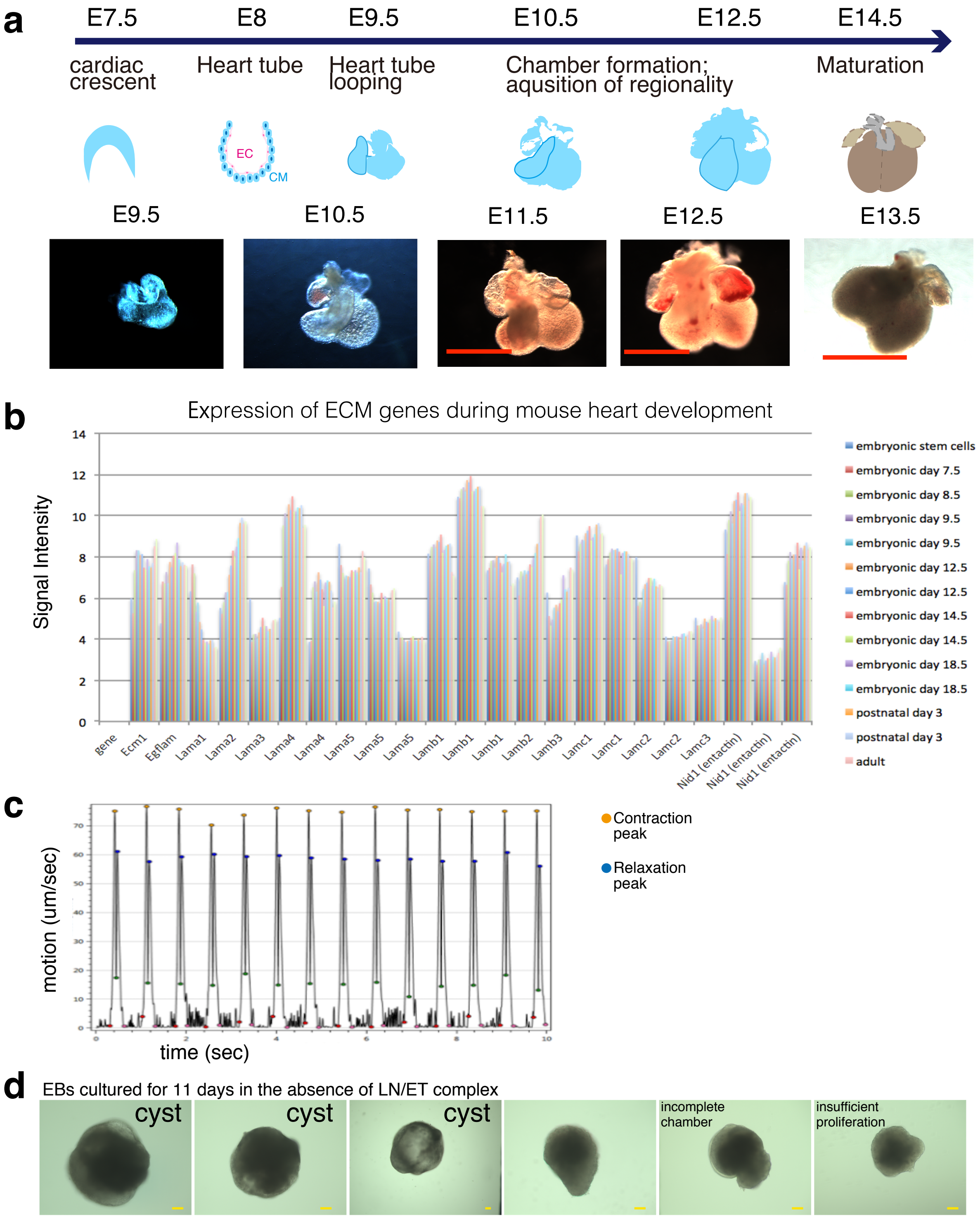


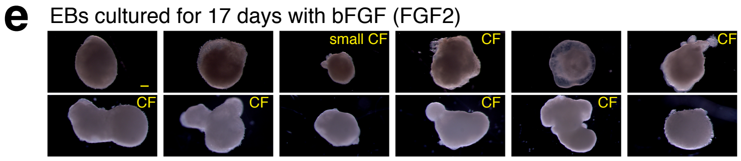


| | Number of chamber formation (CF) | Percentage of CF |
|---|---|---|
| FGF4 | 7 | 77.8 (n=9) |
| bFGF | 7 | 58.3 (n=12) |

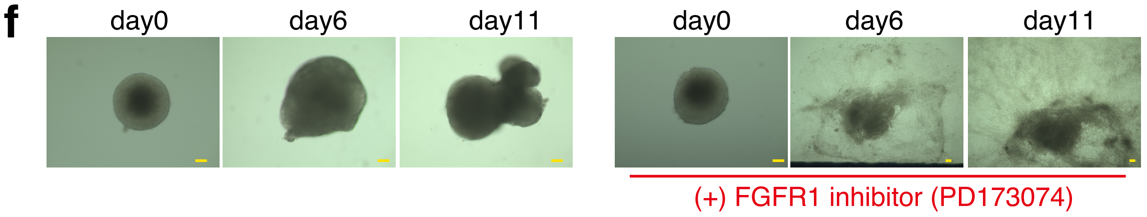

**Supplementary Figure 1. The heart organoid (HO)s mimic mouse heart development. (a)** Heart development during mouse embryogenesis. The cardiac crescent-containing first heart field (FHF) and second heart field (SHF) appeared at E7.5; the structure proceeded to a linear beating heart tube containing outer CMs and inner ECs (E8), became a looping heart tube (E9.5), and subsequently formed a four-chambered heart by E10.5. The hearts were regionally mature at E14.5 (top). Bright field images of embryonic hearts from day 9.5 to 13.5 were presented (bottom). Over n = 2, over two experiments. Scale bar: 1 mm. **(b)** Expression of extracellular matrix (ECM) genes during mouse heart development. We reanalyzed microarray data reported by Li *et al.* (GEO accession No. GDS5003) to investigate the expression of ECM genes in the mouse embryonic heart during development. *Lama1, Lamb1, Lamc1* and *Nid1* (entactin) were dominantly expressed in the embryonic heart. **(c)** Motion vector prediction analysis of the HO cultured for 6 days. The HO showed contractile behavior, with both contraction and relaxation. **(d)** Insufficient proliferation of cardiomyocytes and incomplete chamber formation in the absence of the LN/ET complex (n = 12, two experiments). EBs cultured for 11 days without extracellular matrix failed to form HOs similar to *in vivo* embryonic hearts. Scale bar: 100 $\mu$m. **(e)** Effect of bFGF (FGF2) on HO formation. EBs were cultured with bFGF instead of FGF4 on LN/ET complex (n = 12, two experiments). On culturing day 17, 58% of HOs cultured with bFGF showed the morphological change such as chamber formation. Scale bar: 100 $\mu$m. **(f)** The inhibition of chamber formation during HO culture by FGFR1 inhibitor, PD173074 (n = 9, two experiments). In contrast to the successive formation of HOs in the absence of PD173074 (left), when 100nM PD173074 was continuously added in the culture from day 0, cultured EBs failed to generate HOs (right). Scale bar: 100 $\mu$m.

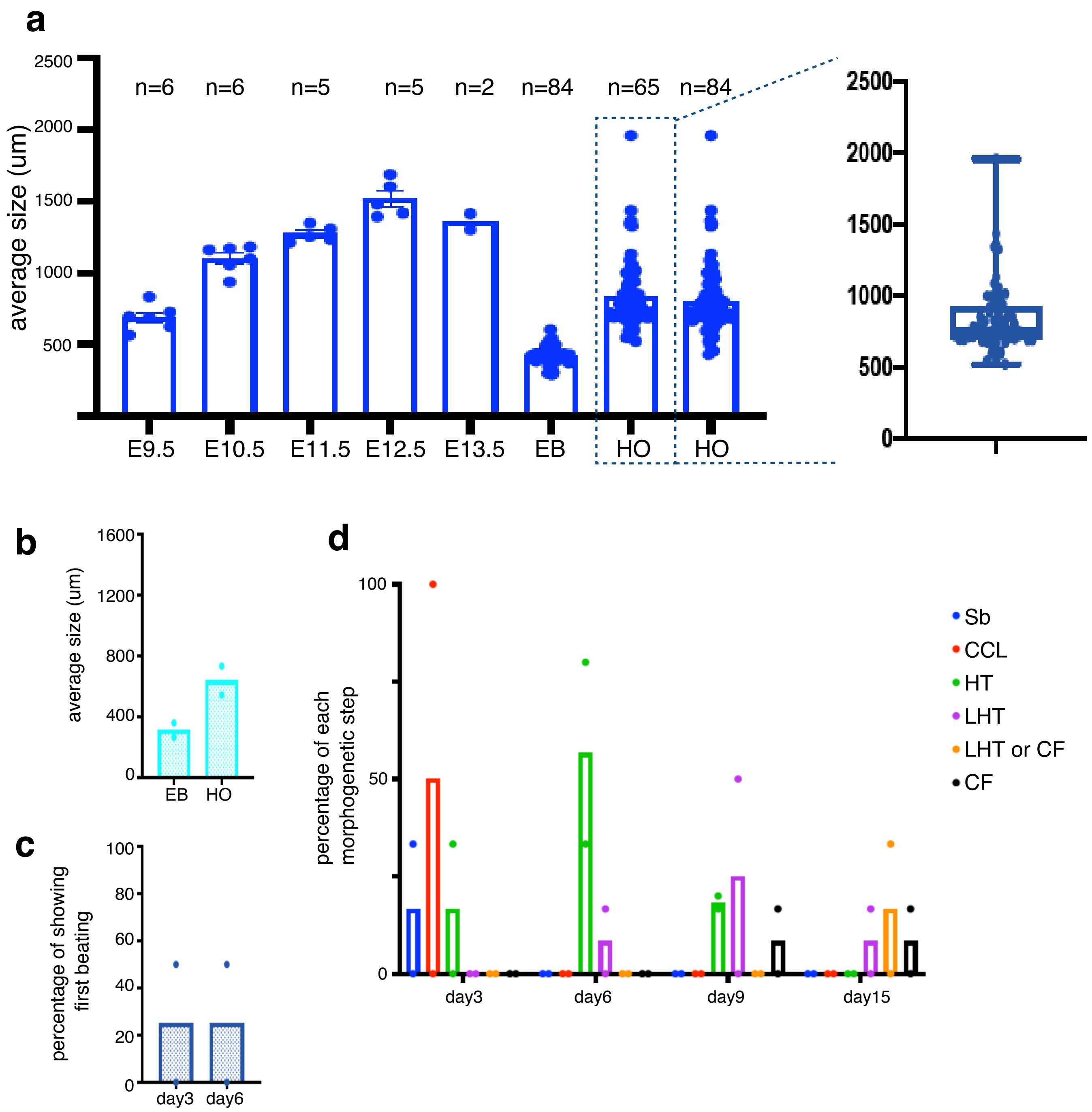


**Supplementary Figure 2. The number of ESCs used for EB culture affects the size, morphology, and function of heart organoid (HO)s during culture. (a)** Size comparison of *in vivo* embryonic hearts from E9.5 to E13.5, EBs prior to culture, and *in vitro* cultured HOs. The HOs derived from the EBs (average, approximately 400 $\mu$m; n = 84) were larger (average, approximately 800 $\mu$m; n = 84) than E9.5 hearts (average, approximately 700 $\mu$m; n = 6). Additionally, HOs exhibiting a looping heart tube or chamber formation (n = 65) were larger than all HOs (n = 84) including HOs at early morphogenetic steps. Box plot indicates the size variation of HOs (n = 65). Data are presented as mean values ± SEM except E13.5 hearts. Box and Whiskers plot: minimum to maximum shows all points. **(b)** Size comparison between EBs prior to culture and *in vitro* cultured HOs derived from small number ESC (500 cells). n = 11, biologically two experiments. **(c)** Only three of 11 (27.3%) *in vitro* cultured HOs derived from small number ESC showed spontaneous beating movement. The number of beating HOs did not increase at day 6. **(d)** Percentages of HOs derived from small number ESC showing specific development-related morphologies according to culture period.Data are presented as mean values in (b), (c), and (d).

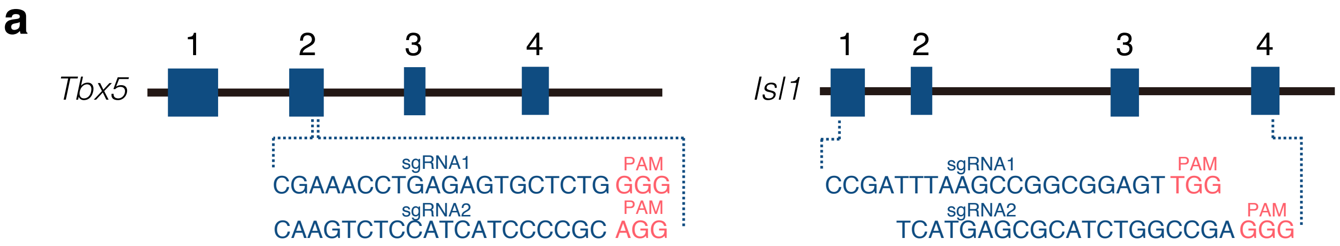


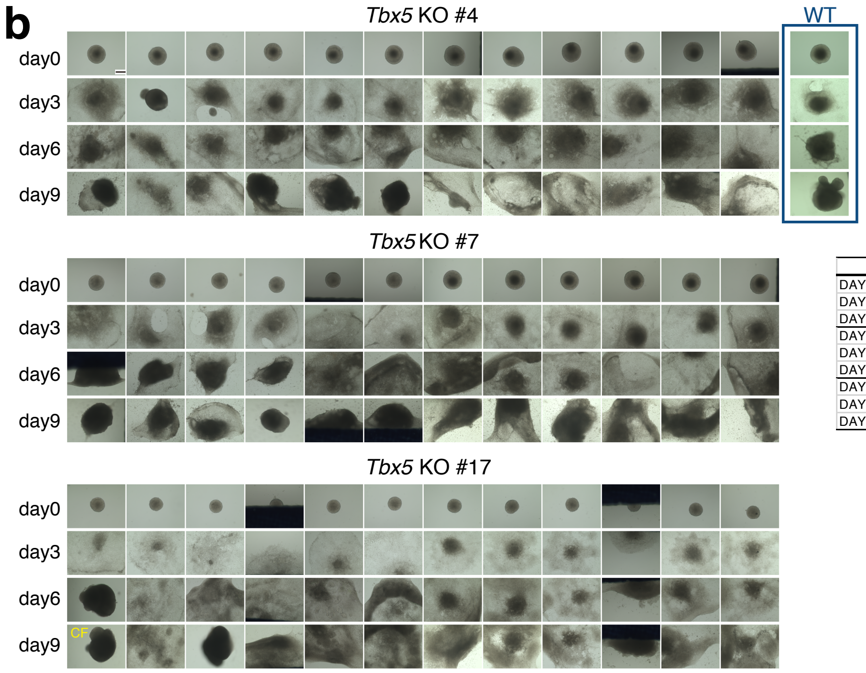


[ percentage of each morphogenetic step ]

| | *Tbx5* KO | SB | CCL | HT | CF |
|---|---|---|---|---|---|
| DAY3 | #4 | 8.3 | 16.7 | 0 | 0 |
| DAY3 | #7 | 0 | 25 | 0 | 0 |
| DAY3 | #17 | 0 | 0 | 0 | 0 |
| DAY6 | #4 | 0 | 8.3 | 0 | 0 |
| DAY6 | #7 | 0 | 33.3 | 0 | 0 |
| DAY6 | #17 | 0 | 8.3 | 8.3 | 0 |
| DAY9 | #4 | 0 | 8.3 | 33.3 | 0 |
| DAY9 | #7 | 0 | 16.7 | 25 | 0 |
| DAY9 | #17 | 0 | 0 | 8.3 | 8.3 |

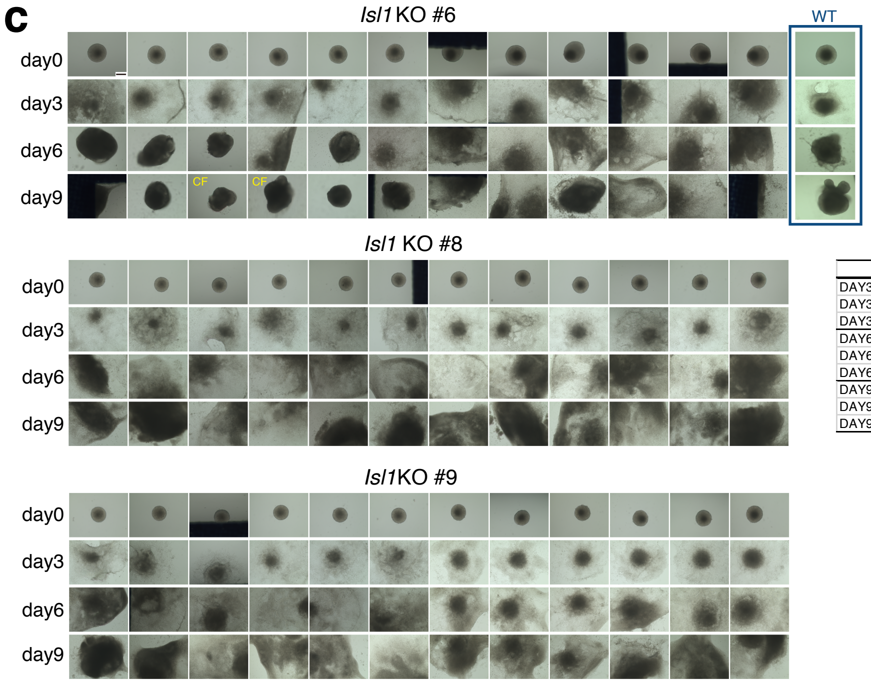


[ percentage of each morphogenetic step ]

| | *Isl1* KO | SB | CCL | HT | CF |
|---|---|---|---|---|---|
| DAY3 | #6 | 0 | 8.3 | 0 | 0 |
| DAY3 | #8 | 0 | 16.7 | 0 | 0 |
| DAY3 | #9 | 0 | 8.3 | 0 | 0 |
| DAY6 | #6 | 8.3 | 25 | 25 | 0 |
| DAY6 | #8 | 0 | 0 | 8.3 | 0 |
| DAY6 | #9 | 0 | 16.7 | 8.3 | 0 |
| DAY9 | #6 | 8.3 | 0 | 33.3 | 16.7 |
| DAY9 | #8 | 0 | 8.3 | 0 | 0 |
| DAY9 | #9 | 0 | 0 | 33.3 | 0 |

**Supplementary Figure 3. Recapitulation of cardiac perturbation in *in vitro* heart organoid (HO) formation using *Tbx5* and *Isl1* KO ESC lines. (a)** Schematic diagram of the strategy to knock out *Tbx5* and *Isl1* in WT ESCs using the CRISPR/Cas system. Two sgRNAs were designed within exon 2 of *Tbx5*, while two sgRNAs within exons 1 and 4 of *Isl1*. **(b)** Three *Tbx5* KO ESC lines were used for generation of HOs. Left: HOs tracking morphological changes over a time course. No chamber formation (CF) was observed in two KO ESC lines (#4 and #9) at day 9 and only one CF occurred in KO ESC line #17 (8.3%, n = 12). WT ESC control experiment was shown in a blue box. Right: a table of the result, showing the percentage of each morphogenetic step during the *Tbx5* KO ESC-derived HO culture. Scale bar: 250 $\mu$m. **(c)** Three of *Isl1* KO ESC lines were used for generation of HOs. Left: HOs tracking morphological changes over a time course. No CF was observed in two KO ESC lines (#8 and #9) and two CF occurred in one KO ESC line (#6) (12%, n = 12) at day 9. WT control is the same as in Supplementary Figure 3b. Right: a table of the result, showing the percentage of each morphogenetic step during the *Isl1* KO ESC-derived HO culture. Scale bar: 250 $\mu$m.

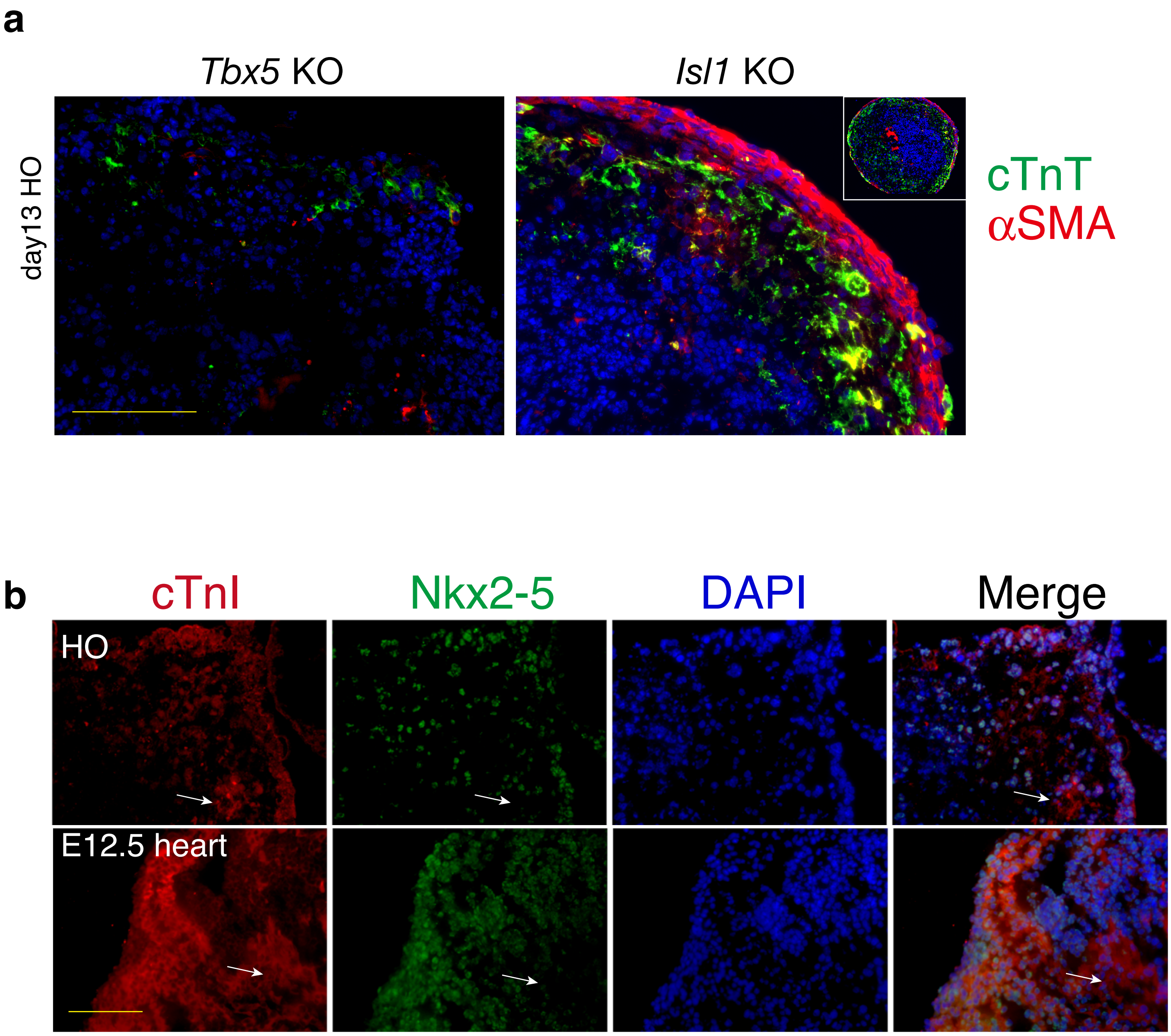


**Supplementary Figure 4. Evaluation of the cell components of heart organoid (HO)s. (a)** Immunostaining to detect αSMA (red)/ cTnT (green) in *Tbx5* KO- and *Isl1* KO-derived HO. Decreased expression of αSMA and cTnT in the *Tbx5* KO-derived HO (n = 14, two experiments) and abnormal location of αSMA, outside the cTnT layer, of the *Isl1* KO-derived HO (n = 10, two experiments) were detected. **(b)** Immunostaining to detect cTnI (red)/Nkx2-5 (green). The population of Nkx2-5 negative and cTnI positive cells (arrows) was detected in the HOs (over n = 5, two experiments).

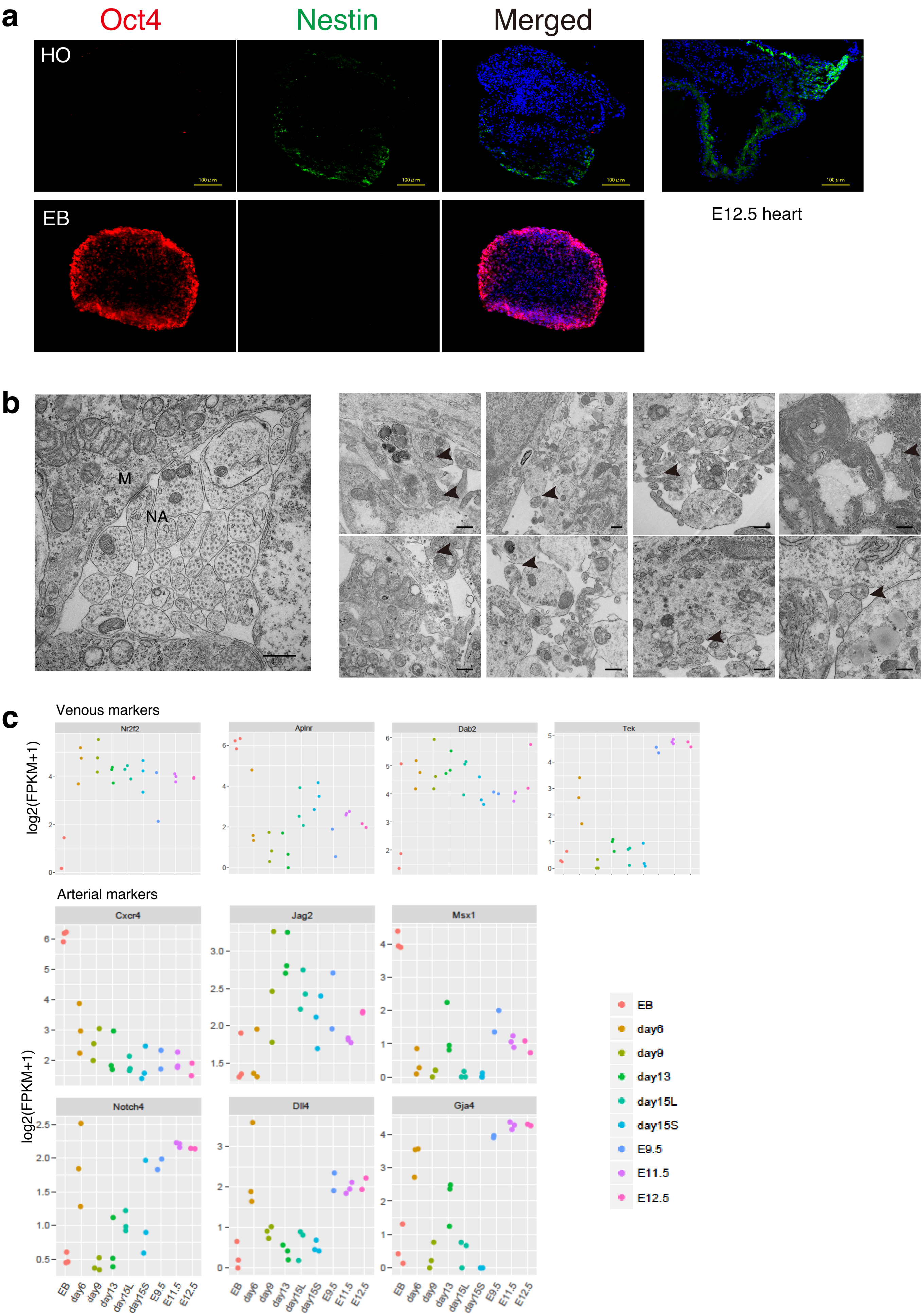
a
Oct4
Nestin
Merged
HO
EB
E12.5 heart
b
M
NA
c
Venous markers
Nr2f2
Aplnr
Dab2
Tek
Arterial markers
Cxcr4
Jag2
Msx1
Notch4
Dll4
Gja4
log2(FPKM+1)
EB
day6
day9
day13
day15L
day15S
E9.5
E11.5
E12.5

**Supplementary Figure 5. Stemness, innervation, and coronary circulation in heart organoid (HO)s. (a)** Immunostaining for Oct3/4 (undifferentiated stem cell marker, red) and Nestin (neural stem cell marker in the fetal heart and brain, green) in a HO (top, Oct3/4 negative and Nestin positive; n = 4 out of n = 17, Oct3/4 negative; n = 17 out of n = 17), an EB (bottom, n = 6), and an embryonic heart from E12.5 (top right). Scale bar: 100 µm. **(b)** Transmission electron microcopy analysis exhibits neuronal innervation in HOs (n = 9 out of n = 11 HOs, over two experiments). Some neuronal axons (NAs) of the naked neuronal axon bundles containing microtubules, small mitochondria, SR, and/or clear vesicles tightly contact the surface of myocytes (M) in the HO cultured for 11 days (left, arrowheads in right panels). Scale bar: 400 nm. **(c)** Gene expression profile of arterial/venous markers for coronary circulation using RNA-seq data. HOs cultured for 6 days (n = 3), for 9 days (n = 3), for 13 days (n = 3), and for 15 days (large size, 15L; small size, 15S; n = 3 each) were used in this analysis. Additionally, EBs (n = 3), E9.5 hearts (n = 2), E11.5 hearts (n = 3), and E12.5 hearts (n = 2) were used. Source data are provided as a source data file.

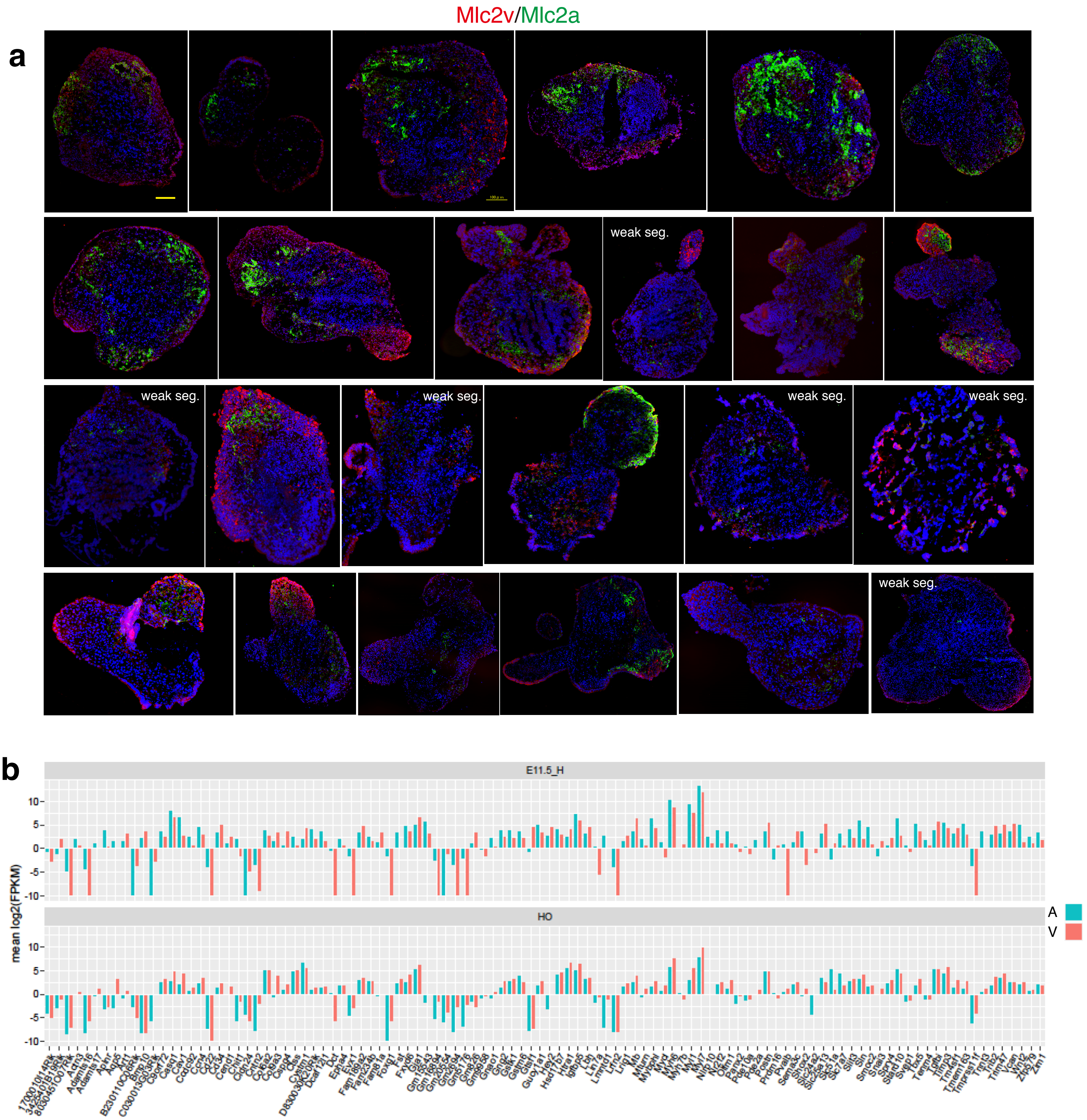


**Supplementary Figure 6. Formation of atrium- and ventricle-like structures. (a)** Immunostaining for Mlc2a (green) and Mlc2v (red) in heart organoid (HO)s (n = 24, over two experiments). Segregated Mlc2a expressions were detected in 18 of HOs and weak segregations (weak seg.) were observed in 6 of HOs. Scale bar: 100 $\mu$m. **(b)** Gene expression profile of atrium- and ventricle-like structures of HOs and atria and ventricles of E11.5 hearts using RNA-seq data. HOs cultured for 13 days (n = 3) were surgically dissected to each atrium-like part and ventricle-like part for analysis. Bar plot showed similar gene expression profiles of atrium- and ventricle-like structures of HOs and atria and ventricles of E11.5 hearts using RNA-seq data based on the differentially expressed genes (106 genes) between atria and ventricles of *in vivo* embryonic hearts. See also Fig. 8d and e, top. Source data are provided as a source data file.

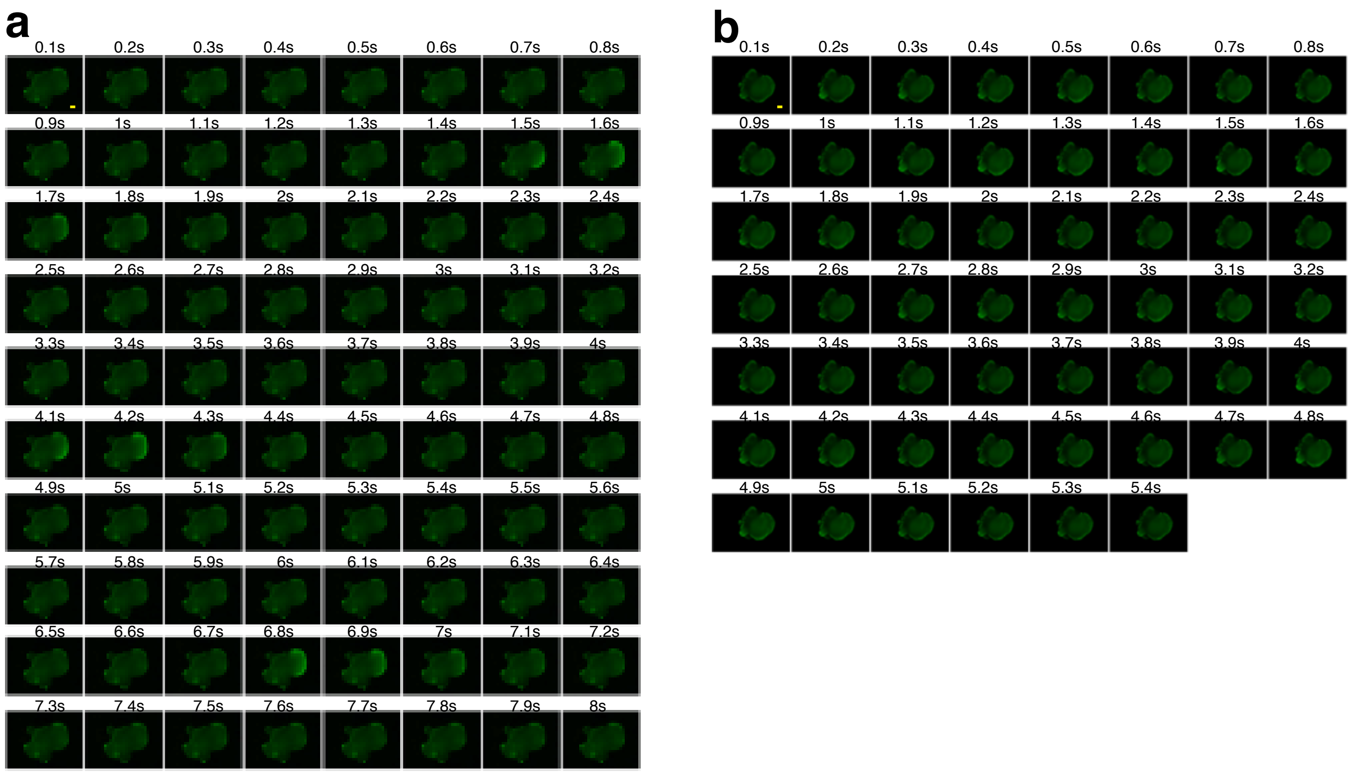

**Supplementary Figure 7. Sequential images of calcium transients in heart organoid (HO)s. (a), (b)** Calcium oscillations in HOs (n = 7, two experiments) were analyzed by calcium binding fluorescent dyes, Fluo8 AM or Fluo8 AM/F127. Image stamps of sequential images by 10 frames per second (80 (**a**) and 54 sequential images (**b**)). See also Fig. 9a and Supplementary Movie 5. Scale bar: 100 $\mu$m.
